# Efficacy and safety of acupuncture for coronary microvascular disease: study protocol for a pilot randomized controlled trial

**DOI:** 10.3389/fcvm.2026.1843651

**Published:** 2026-07-17

**Authors:** Linghao Meng, Houfang Ma, Di Sun, Kun Xia, Shuhan Zhao, Songyan Bai, Wenzeng Zhu, Shuai Shi, Limei Zhang

**Affiliations:** 1 Guang'anmen Hospital, China Academy of Chinese Medical Sciences, Beijing, China; 2 China Center for Evidence-Based Traditional Chinese Medicine, China Academy of Chinese Medical Sciences, Beijing, China

**Keywords:** acupuncture, coronary microvascular disease, ischemia with non-obstructive coronary arteries, randomized controlled trial, sham acupuncture, study protocol

## Abstract

**Background:**

Coronary microvascular disease (CMVD), particularly ischemia with non-obstructive coronary arteries (INOCA)-related CMVD, is associated with recurrent angina, impaired quality of life, and an increased risk of adverse cardiovascular outcomes. Although recognition of this condition is increasing, treatment options remain limited, and many patients continue to have symptoms despite conventional medical therapy. Acupuncture has shown potential as an adjunctive therapy for angina-related symptoms and other cardiovascular conditions. However, high-quality evidence for INOCA-related CMVD remains scarce. This trial aims to evaluate the efficacy and safety of acupuncture as an adjunct to conventional medical therapy in patients with INOCA-related CMVD.

**Methods:**

This prospective, single-center, randomized, sham-controlled, parallel-group pilot trial will recruit 60 participants with INOCA-related CMVD at Guang'anmen Hospital, China Academy of Chinese Medical Sciences, Beijing, China. Eligible participants will be randomly assigned in a 1:1 ratio to an acupuncture group or a sham acupuncture group. Both groups will continue guideline-directed conventional medical therapy. The acupuncture group will receive true acupuncture at Dabao (SP21), Neiguan (PC6), Danzhong (CV17), Xinshu (BL15), and Geshu (BL17), while the sham group will receive non-penetrating sham acupuncture at the same acupoints with the same schedule. Treatment will be given three times weekly for 4 consecutive weeks, followed by a 4-week follow-up. The primary outcome is the response rate at week 4, defined as the proportion of participants with an increase of at least 5 points in the Seattle Angina Questionnaire summary score from baseline. Secondary outcomes include changes in SAQ scores, angina attack frequency, EuroQol 5-Dimension 5-Level questionnaire, traditional Chinese medicine syndrome score, nitric oxide, endothelin-1, and high-sensitivity C-reactive protein. Additional assessments include Clinical Global Impression–Improvement, expectancy and credibility assessments, and blinding assessment. Safety evaluation includes monitoring adverse events and serious adverse events throughout the trial.

**Discussion:**

This pilot trial will provide preliminary evidence on the safety and potential efficacy of acupuncture for INOCA-related CMVD and may help clarify its effects on angina-related health status, quality of life, and selected endothelial and inflammation-related markers. The findings are expected to inform future larger multicenter confirmatory trials.

**Clinical Trial Registration:**

International Traditional Medicine Clinical Trial Registry (http://itmctr.ccebtcm.org.cn/), Identifier ITMCTR2026000515.

## 1 Introduction

Coronary microvascular disease (CMVD) is a clinical syndrome caused by structural and/or functional abnormalities of the coronary microcirculation in the absence of obstructive epicardial coronary artery disease (1, 2). Its major pathophysiological mechanisms include endothelial dysfunction, impaired vasomotor regulation, and reduced microvascular perfusion. In recent years, CMVD has been increasingly recognized as an important phenotype within chronic coronary syndromes and a major mechanism underlying persistent angina in patients with angina and non-obstructive coronary arteries (ANOCA/INOCA) (3, 4).

CMVD is common in patients with ANOCA/INOCA, particularly in women, although its prevalence varies according to diagnostic modality and threshold definitions (5, 6). More importantly, CMVD is not a benign condition. Patients with coronary microvascular dysfunction often experience recurrent chest pain, impaired functional capacity, and reduced quality of life, and may also face an increased long-term risk of adverse cardiovascular outcomes (7, 8). Despite growing awareness of this condition, its clinical management remains challenging. Although recent guidelines have emphasized early recognition and mechanism-based treatment, access to specialized diagnostic testing is still limited, and many patients continue to have persistent symptoms despite standard anti-anginal therapy and risk-factor control (2, 9, 10).

Acupuncture has shown potential as a non-pharmacological adjunctive therapy for cardiovascular diseases, particularly chronic stable angina. Randomized trials and evidence syntheses suggest that acupuncture may help relieve angina symptoms and improve patient-reported outcomes (11–14). Emerging reviews on CMVD and traditional Chinese medicine further suggest that acupuncture may exert beneficial effects through modulation of autonomic function, endothelial function, inflammation, and microcirculatory regulation (15–17). However, the current evidence for acupuncture specifically in CMVD remains limited. Most published studies are small, single-center, and methodologically heterogeneous, and important shortcomings remain in diagnostic standardization and outcome assessment (15, 18–20).

Therefore, a rigorously designed randomized controlled trial is needed to evaluate the efficacy and safety of acupuncture for INOCA-related CMVD. In the context of contemporary ANOCA/INOCA frameworks, the target population of this trial is symptomatic INOCA-related CMVD, corresponding mainly to coronary microvascular dysfunction characterized by adenosine-mediated impaired vasodilation and/or abnormal vasoconstriction of the coronary microvasculature, rather than epicardial vasospastic angina or asymptomatic coronary microvascular dysfunction. The present study is designed as a prospective, single-center, randomized, sham-controlled exploratory trial to assess whether acupuncture, in addition to conventional therapy, can improve angina-related health status, quality of life, and endothelial and inflammatory markers in this population. By incorporating validated patient-reported outcomes, such as the Seattle Angina Questionnaire, together with biomarker-based evaluation, this trial is expected to provide more standardized preliminary evidence for acupuncture in INOCA-related CMVD and to inform the design of future larger multicenter studies (21).

## 2 Methods and analysis

### 2.1 Study design

This is a prospective, single-center, parallel-group, randomized, sham-controlled exploratory trial. The protocol was prepared in accordance with the SPIRIT 2025 statement (22), with the corresponding checklist provided in Supplementary Table S1, and the acupuncture intervention is reported with reference to the revised STRICTA recommendations (23), as detailed in Supplementary Table S2. Sixty eligible participants will be randomly assigned in a 1:1 ratio to the acupuncture group or the sham acupuncture group.

Each participant will be followed for 8 weeks, including baseline assessment within 24 h after enrollment, 4 weeks of treatment, and 4 weeks of post-treatment follow-up. The primary objective is to evaluate the efficacy and safety of acupuncture as an adjunct to conventional therapy for INOCA-related CMVD and to explore its potential effects on angina-related health status, quality of life, endothelial function, and inflammation-related biomarkers.

**Figure 1:**
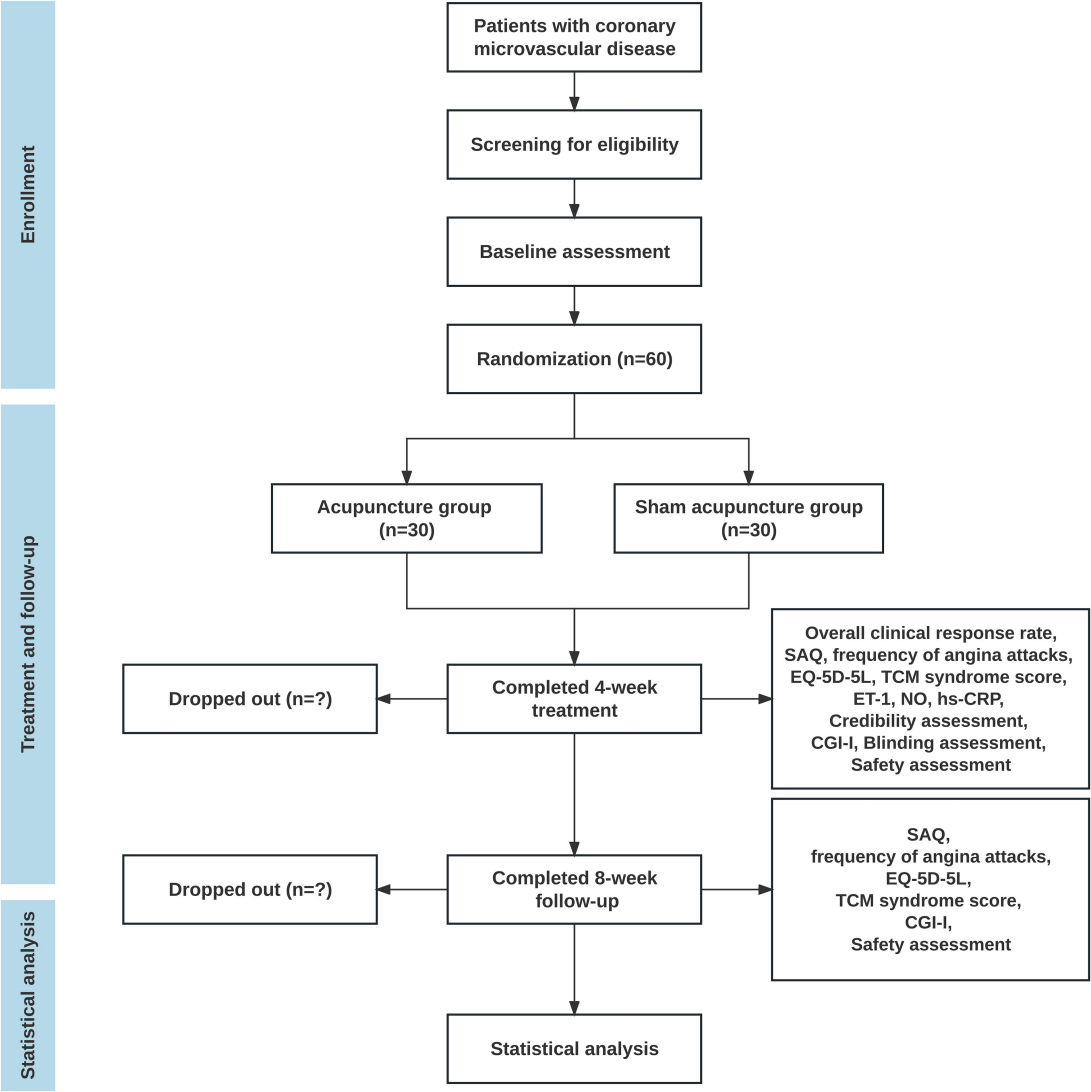
The study flowchart. SAQ, Seattle Angina Questionnaire; EQ-5D-5L, EuroQol 5-Dimension 5-Level questionnaire; TCM, traditional Chinese medicine; ET-1, endothelin-1; NO, nitric oxide; hs-CRP, high-sensitivity C-reactive protein; CGI-I, Clinical Global Impression-Improvement.

This study protocol is version 1.0. It has been approved by the Ethics Committee of Guang'anmen Hospital, China Academy of Chinese Medical Sciences, and was registered with the International Traditional Medicine Clinical Trial Registry on March 13, 2026 (registration number: ITMCTR2026000515). The trial will be conducted in accordance with the Declaration of Helsinki. Eligible participants will be enrolled voluntarily and will provide written informed consent before any study-specific procedure. The study flowchart and the schedule of enrollment, interventions, and assessments are shown in Figure 1 and Table 1.

**Table 1:** Study schedule.

Stage	Trial period			
	Enrollment	Allocation	Treatment	Follow-up
Timepoint	−1d	0d	4w	8w
Enrollment				
Eligibility screening	×			
Informed consent	×			
Baseline assessment		×		
Randomization		×		
Interventions				
Acupuncture plus conventional therapy		←--------------→		
Sham acupuncture plus conventional therapy		←--------------→		
Assessments				
Demographic and baseline clinical characteristics		×		
Overall clinical response rate			×	
SAQ		×	×	×
Angina attack frequency		×	×	×
EQ-5D-5L		×	×	×
TCM syndrome score		×	×	×
Laboratory tests (NO, ET-1, hs-CRP)		×	×	
Patients’ expectation for acupuncture		×		
Credibility assessment		×	×	
CGI-I			×	×
Blinding assessment			×	
Safety assessment and adverse events		×	×	×

d, day; w, week; SAQ, Seattle Angina Questionnaire; EQ-5D-5L, EuroQol 5-Dimension 5-Level questionnaire; TCM, Traditional Chinese Medicine; NO, nitric oxide; ET-1, endothelin-1; hs-CRP, high-sensitivity C-reactive protein; CGI-I, Clinical Global Impression–Improvement.

### 2.2 Participant screening

#### 2.2.1 Diagnostic criteria

##### 2.2.1.1 Diagnostic criteria for INOCA-related CMVD

According to the Chinese expert consensus on the diagnosis and treatment of coronary microvascular diseases (2023 edition) (1), INOCA-related CMVD will be diagnosed when all of the following conditions are satisfied:

1. Typical clinical symptoms are present.
2. At least one objective indicator of myocardial ischemia is documented, including typical exertional or spontaneous chest pain accompanied by ST-segment depression on electrocardiography; reversible myocardial perfusion defects on stress single-photon emission computed tomography (SPECT); reduced coronary flow reserve (CFR <2.0) assessed by Doppler echocardiography; reduced myocardial perfusion reserve index (MPRI <2.0) on cardiac magnetic resonance imaging (CMR); or metabolic evidence of myocardial ischemia on positron emission tomography (PET).
3. Coronary angiography shows normal coronary arteries, irregular vessel walls, or luminal stenosis of <50%.
4. In patients with a high clinical suspicion of CMVD despite CFR ≥ 2.0, an intracoronary acetylcholine provocation test may be performed under close monitoring when clinically indicated. The presence of epicardial coronary spasm during acetylcholine testing will lead to exclusion. In contrast, reproduction of angina symptoms and ischemic ST-T changes in the absence of epicardial coronary spasm will be considered compatible with microvascular vasomotor dysfunction.
5. Non-cardiac chest pain and other cardiac diseases are excluded, including vasospastic angina, cardiomyopathy, myocarditis, and valvular heart disease.

##### 2.2.1.2 Traditional Chinese medicine syndrome differentiation criteria

The diagnostic criteria for qi deficiency and blood stasis syndrome will be based on the Guidelines for the Diagnosis and Treatment of Coronary Microvascular Disease in Traditional Chinese Medicine (24).

1. Primary symptom: chest pain or chest tightness, usually triggered or aggravated by exertion.
2. Secondary symptoms: ① shortness of breath and fatigue;② lassitude and reluctance to speak;③ palpitations and spontaneous sweating;④ pale or dusky complexion.
3. Tongue manifestation: enlarged, pale-dark tongue.
4. Pulse manifestation: deep and choppy pulse.

A diagnosis will be made when at least one primary symptom is present, together with two or more secondary symptoms, in combination with the corresponding tongue and pulse manifestations.

#### 2.2.2 Inclusion criteria

Participants will be eligible for inclusion if they meet all of the following criteria:

1. Aged 18 to 80 years, regardless of sex;
2. Meeting the Western medicine diagnostic criteria for INOCA-related CMVD;
3. Meeting the traditional Chinese medicine diagnostic criteria for qi deficiency and blood stasis syndrome;
4. Having a relatively stable medication regimen during the month before enrollment and being able to complete acupuncture treatment and follow-up;
5. Willing to accept random allocation, able to understand the study procedures, and providing written informed consent.

#### 2.2.3 Exclusion criteria

Participants will be excluded if they meet any of the following criteria:

1. Acute coronary syndrome or coronary revascularization within the previous 3 months; significant valvular heart disease, cardiomyopathy, or heart failure of New York Heart Association class III-IV;
2. Definite or clinically suspected epicardial vasospastic angina, based on clinical history, electrocardiographic findings during symptoms, angiographic evidence, or acetylcholine provocation testing when available;
3. Severe hepatic or renal dysfunction, active bleeding, or coagulation disorders;
4. Pregnancy or lactation;
5. Contraindications to acupuncture, such as local infection, severe skin disease, or implanted devices located in areas at risk during needling;
6. Allergy or contraindication to background medical therapy.

### 2.3 Recruitment process

All recruiters and study personnel will receive standardized training before trial initiation. Recruitment advertisements will be posted in outpatient clinics and wards of Guang'anmen Hospital, and recruitment information will also be disseminated through the hospital's official WeChat public platform.

### 2.4 Written informed consent

Before study initiation, recruiters will provide potential participants with a detailed explanation of the possible benefits, risks, and discomforts associated with participation. After reviewing the paper informed-consent form, participants who agree to enroll will sign the form in duplicate, with one copy retained by the investigator and the other by the participant or the participant's legal representative. Contact information will be collected to facilitate communication during treatment and follow-up. Participants who decline participation, or who withdraw at any time, will not be affected with respect to routine medical care or any other rights and interests.

### 2.5 Participant adherence

To improve adherence, participants will receive education on the epidemiology, diagnosis, and treatment of CMVD, together with a clear explanation of the study background and the potential benefits and risks of acupuncture. Investigators will maintain close contact with participants and, when appropriate, family members, and will provide reminders for treatment sessions and follow-up visits. All protocol-required laboratory tests, examinations, and questionnaire assessments will be provided free of charge. For participants who discontinue the study, treatment status and reasons for withdrawal will be documented to support adherence assessment.

### 2.6 Randomization, allocation concealment, and blinding

An independent statistician will generate a simple 1:1 randomization sequence without stratification using SAS 9.4 and a prespecified random seed. Considering the exploratory nature and relatively small sample size of this pilot trial, stratification by sex, age, or baseline SAQ score will not be performed. Allocation will be concealed in sequentially numbered, sealed, opaque envelopes prepared and kept by staff members not involved in recruitment, treatment, or outcome assessment. Each envelope will be opened in numerical order only after eligibility has been confirmed and baseline assessment has been completed. Participants, outcome assessors, and statisticians will remain blinded to group assignment. Outcome assessors will not participate in treatment delivery or allocation procedures. The dataset provided to the statistician will use coded group labels, and the allocation list will not be released until the primary analysis has been completed. Acupuncturists cannot be blinded because of the nature of the intervention. Emergency unblinding will be allowed only when knowledge of the allocated intervention is essential for clinical management of a serious adverse event or another urgent medical situation. Any emergency unblinding will be authorized by the principal investigator, documented in detail, including the reason, date, personnel involved, and participant status, and reported to the ethics committee when required.

To minimize visual cues during treatment, participants will be positioned so that the needling procedure is not in their direct line of sight. For acupoints on the forearm and anterior chest, an opaque drape or screen will be used where feasible, and participants will be instructed not to observe needle insertion or removal. For back acupoints, participants will be unable to directly observe the procedure. The same adhesive pads, treatment setting, participant position, verbal instructions, and session duration will be used in both groups. The sham needles will have a similar external appearance to real needles but will be blunt-tipped and non-penetrating. These procedures are intended to reduce visual and contextual differences between groups.

### 2.7 Interventions

Participants in both groups will continue conventional medical therapy recommended by current guidelines for chronic coronary syndromes and INOCA, including anti-anginal agents for symptom relief and preventive treatment for cardiovascular risk-factor control (2). To minimize potential bias related to pharmacotherapy, all participants will be required to have a relatively stable medication regimen for at least 1 month before enrollment. Guideline-directed conventional medical therapy will be continued in both groups throughout the trial. Medication changes during the study will be avoided unless clinically necessary. Any change in drug type, dose, timing, or indication will be recorded in detail, and substantial medication changes will be considered in sensitivity or exploratory analyses where appropriate.

#### 2.7.1 Acupuncture group

**Figure 2:**
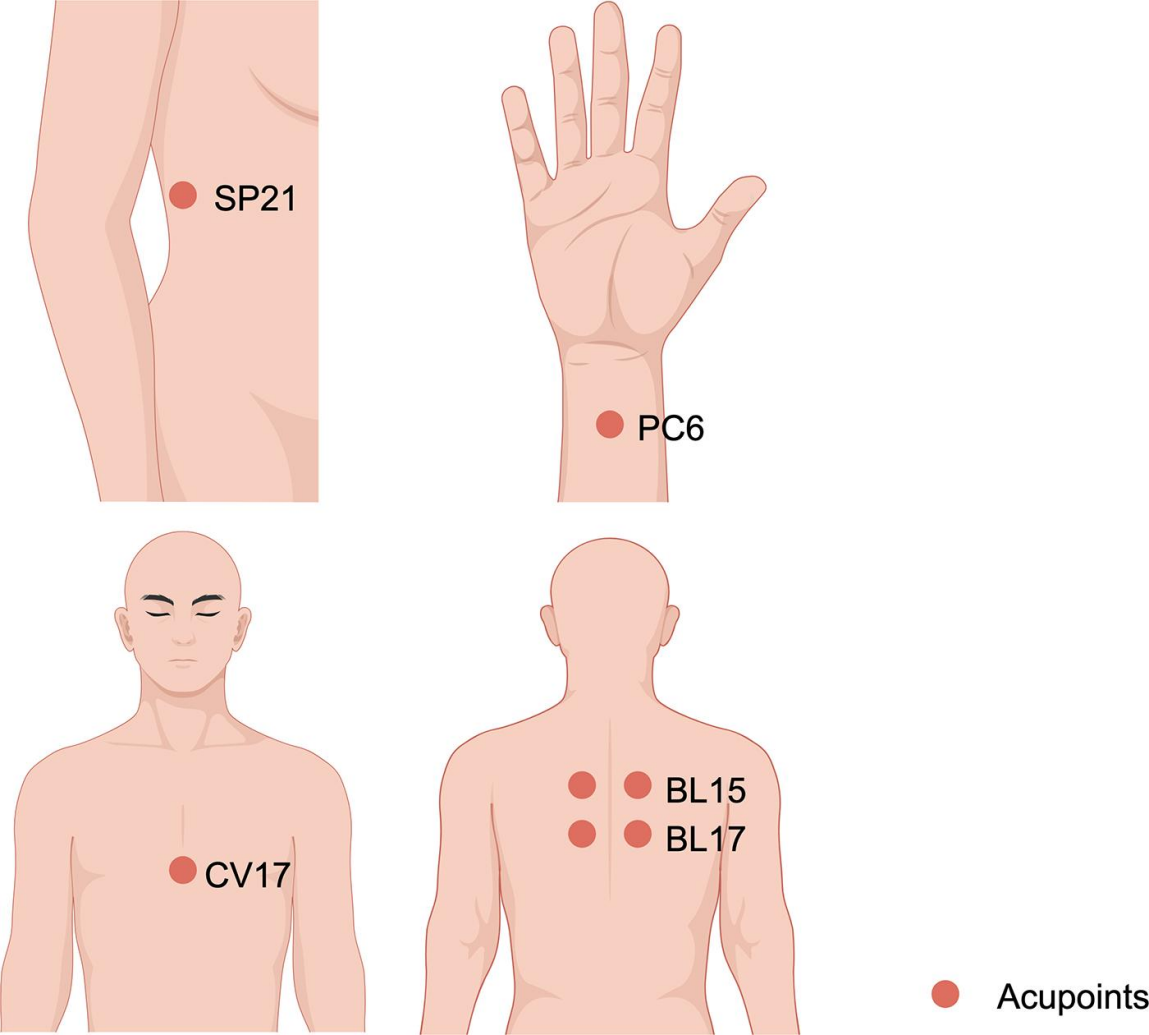
Locations of the acupoints.

Participants in the acupuncture group will receive true acupuncture in addition to conventional drug therapy. The prespecified acupoints are Dabao (SP21), Neiguan (PC6), Danzhong (CV17), Xinshu (BL15), and Geshu (BL17). The nomenclature and locations of these acupoints follow the national standard GB/T 12346–2021 (25). Their surface locations and detailed information are shown in Figure 2 and Table 2.

**Table 2:** Locations of the acupoints used in this study.

Acupoint	Location
Dabao (SP21)	In the lateral thoracic region, in the 6th intercostal space, on the midaxillary line.
Neiguan (PC6)	In the anterior forearm region, 2 cun proximal to the palmar wrist crease, between the tendons of palmaris longus and flexor carpi radialis.
Danzhong (CV17)	In the anterior thoracic region, on the anterior median line, at the level of the 4th intercostal space.
Xinshu (BL15)	In the upper back region, at the level of the lower border of the spinous process of the 5th thoracic vertebra, 1.5 cun lateral to the posterior median line.
Geshu (BL17)	In the upper back region, at the level of the lower border of the spinous process of the 7th thoracic vertebra, 1.5 cun lateral to the posterior median line.

During each session, participants will sit comfortably with the area below the elbows and the chest, hypochondriac region, and back exposed. After routine skin disinfection, adhesive pads will be placed over the selected acupoints. Disposable sterile stainless-steel filiform needles (0.30 mm × 40 mm; Huatuo, Suzhou, China) will then be inserted through the pads to an appropriate depth sufficient to elicit deqi. After insertion, manual needle manipulation will be performed to elicit deqi, typically described as soreness, numbness, distension, or heaviness. No specific tonification or sedation technique will be prespecified. During the 20 min retention period, no additional routine manipulation will be performed unless required to maintain deqi according to the standardized procedure. Treatment will be administered three times weekly, every other day, with 20 min of needle retention per session for 4 consecutive weeks. Further details of the intervention, including the number of needle insertions, unilateral or bilateral point use, depth of insertion, response sought, and needle stimulation, are provided in Supplementary Table S2.

#### 2.7.2 Sham acupuncture group

Participants in the sham acupuncture group will receive sham acupuncture in addition to conventional drug therapy, using the same acupoints and treatment schedule as the acupuncture group. After routine disinfection and placement of adhesive pads, a blunt-tipped sham needle (0.30 mm × 25 mm; Huatuo, Suzhou, China) will be applied so that it passes through the pad and touches the skin surface without penetrating the skin or subcutaneous tissue, and deqi will not be sought. The sham procedure will be matched with true acupuncture in terms of acupoint location, participant position, treatment environment, treatment frequency, session duration, and overall treatment course. Contact pressure will be kept light and as consistent as possible across sessions. The needle-withdrawal procedure will be identical to that in the acupuncture group.

All procedures will be performed by licensed traditional Chinese medicine practitioners certified by the National Health Commission of the People's Republic of China, each with at least 2 years of clinical experience in acupuncture.

### 2.8 Outcome measures

#### 2.8.1 Primary outcome

The primary outcome is the overall clinical response rate at week 4, defined as the proportion of participants who achieve an increase of at least 5 points in the Seattle Angina Questionnaire summary score (SAQ-SS) from baseline. The Seattle Angina Questionnaire (SAQ) is a disease-specific 19-item instrument comprising five domains—physical limitation, angina stability, angina frequency, treatment satisfaction, and quality of life—with each domain scored from 0 to 100; higher scores indicate better angina-related health status (26). The SAQ-SS is calculated as the mean of the physical limitation, angina frequency, and quality-of-life domain scores (19). Consistent with previous studies, an increase of 20 points or more will be considered a large improvement, an increase of 5–19 points a clinically meaningful improvement, and an increase of less than 5 points, no change, or deterioration as no response (27, 28). The responder rate will be calculated as the number of participants with an SAQ-SS increase of at least 5 points divided by the total number of participants.

#### 2.8.2 Secondary outcomes

1. Seattle Angina Questionnaire (SAQ) scores:

The SAQ-SS and individual SAQ domain scores will be assessed at baseline, week 4, and week 8 to evaluate continuous changes in angina-related symptoms and health status.

2. Angina attack frequency:

Angina episodes will be continuously recorded and evaluated using an angina diary from baseline through the end of the 8-week follow-up period. The diary will document the onset time, end time, duration of each episode, pain intensity measured by the visual analogue scale (VAS), accompanying symptoms, medication use, nitroglycerin dosage, and symptom relief.

3. EuroQol five-dimension five-level questionnaire (EQ-5D-5L):

The EQ-5D-5L will be assessed at baseline, week 4, and week 8. This instrument includes five dimensions: mobility, self-care, usual activities, pain/discomfort, and anxiety/depression, each rated on a five-level scale (29). In addition, the EQ visual analogue scale (EQ-VAS) will be recorded to reflect the participant's self-rated health status on the day of assessment. The EQ-5D-5L will be used to evaluate the impact of acupuncture on overall health status and quality of life.

4. Traditional Chinese medicine (TCM) syndrome score:

The TCM syndrome score will be assessed at baseline, week 4, and week 8. The scoring system includes the following major symptoms: chest pain and chest tightness, each scored as 0, 2, 4, or 6 points; and the following minor symptoms: palpitations, shortness of breath, fatigue, spontaneous sweating, and dark complexion, each scored as 0, 1, 2, or 3 points. Tongue manifestation, tongue coating, and pulse condition will each be scored as 0 or 1 point. A higher total score indicates more severe manifestations of qi deficiency and blood stasis syndrome. The total TCM syndrome score and its change from baseline will be compared between the two groups to evaluate the effect of acupuncture on qi deficiency and blood stasis syndrome.

5. Endothelial function and inflammation-related biomarkers:

Blood samples will be collected at baseline and week 4 for measurement of nitric oxide (NO), endothelin-1 (ET-1), and high-sensitivity C-reactive protein (hs-CRP). These markers will be used to reflect changes in endothelial vasomotor function and inflammatory status and to explore the potential biological effects of acupuncture in CMVD (30, 31). These biomarkers are intended to explore short-term treatment-related changes in endothelial function and inflammatory status during the intervention period.

#### 2.8.3 Additional outcomes

Additional outcomes in this study will include baseline expectancy assessment, credibility assessment of acupuncture therapy, the Clinical Global Impression–Improvement scale (CGI-I), and participant blinding assessment.

Baseline expectancy will be assessed before the first treatment session. Credibility of acupuncture will be evaluated at baseline and again at week 4 (32). The CGI-I will be assessed at week 4 and at the end of follow-up to capture participants' global perception of change relative to baseline (33). Participant blinding assessment will be completed within 5 min after the final treatment session at week 4. Participants will be asked to guess whether they received true acupuncture, sham acupuncture, or were unsure. The success of blinding will be evaluated by comparing the distribution of allocation guesses between groups (34).

Because objective evidence of myocardial ischemia is required for inclusion, this study focuses on INOCA-related CMVD rather than broader ANOCA or asymptomatic coronary microvascular dysfunction. Accordingly, the symptom- and quality-of-life-related outcomes, including SAQ scores, angina attack frequency, EQ-5D-5L, and CGI-I, will be evaluated in a symptomatic INOCA-related CMVD population.

### 2.9 Safety evaluation

An adverse event will be defined as any unfavorable medical occurrence during the trial, regardless of its causal relationship with the intervention. A serious adverse event will be defined as an event resulting in death, life-threatening condition, hospitalization or prolongation of hospitalization, persistent or significant disability, or any other medically important event. The severity of adverse events will be graded as mild, moderate, or severe, with reference to CTCAE version 5.0 where applicable. Cardiovascular adverse events, including newly developed arrhythmia, heart failure, and acute myocardial infarction, as well as acupuncture-related adverse events such as syncope, intolerable pain, local hematoma, and infection, will be recorded in detail. The type, onset and resolution time, severity, management, and outcome of each adverse event will be documented in the case report form.

Preventive measures for acupuncture-related adverse events will include treatment by licensed practitioners, use of sterile disposable needles, routine skin disinfection, appropriate participant positioning, and close observation during and after treatment. Before each session, participants will be advised to have an appropriate light meal, obtain adequate rest, and avoid fasting, fatigue, or excessive emotional stress. During treatment, practitioners will closely observe the participant's facial expression, sweating, dizziness, palpitations, nausea, and other symptoms. Local hematoma will be managed by compression and observation; vasovagal syncope will be managed by immediate cessation of needling, needle removal, appropriate positioning, vital-sign monitoring, and supportive care; suspected infection will be evaluated and treated promptly; and intolerable pain will lead to cessation or adjustment of the procedure.

Any serious adverse event will be reported immediately to the principal investigator and to the ethics committee within 24 h or according to institutional requirements. Whether the participant continues, temporarily suspends, or withdraws from the trial will be determined by the investigator according to clinical judgment and participant safety. The incidence of adverse events in each group will be summarized after completion of the 8-week study period.

### 2.10 Sample size

Given the pilot nature of this trial and the limited high-quality randomized evidence on acupuncture specifically for INOCA-related CMVD, a reliable effect size for a formal confirmatory sample size calculation is not available. Previous acupuncture studies in angina-related conditions support the clinical relevance of SAQ-based outcomes, but their populations, diagnostic criteria, and interventions are not sufficiently comparable to provide a directly transferable effect-size estimate for this trial. Therefore, the sample size was chosen to support feasibility assessment and to obtain preliminary estimates of variability and effect size, especially for SAQ-SS changes and the week 4 responder rate. On the basis of commonly cited recommendations for pilot studies, 25 participants per group was considered appropriate to assess feasibility, estimate variance, and obtain preliminary effect-size information (35, 36). Allowing for an anticipated dropout rate of approximately 20%, 60 participants (30 per group) will be recruited (37). The results of this pilot trial will be used to inform the design and sample-size estimation of future multicenter confirmatory randomized trials.

### 2.11 Statistical analysis

All analyses will be performed using SPSS version 27.0, and figures will be generated using GraphPad Prism version 10. The primary analysis will follow the intention-to-treat principle, with per-protocol analysis conducted as sensitivity analysis. Safety analysis will include all participants who receive at least one treatment session. Baseline characteristics will be summarized by group using appropriate descriptive statistics. Continuous variables will be presented as mean ± standard deviation or median with interquartile range, as appropriate, and categorical variables as frequencies and percentages. Baseline comparability between groups will be assessed descriptively and, where appropriate, using *t*-test or Mann–Whitney *U*-test for continuous variables and chi-square test or Fisher's exact test for categorical variables. The primary outcome, overall clinical response rate at week 4, will be compared between groups using logistic regression, with results reported as odds ratios and 95% confidence intervals. Repeated continuous secondary outcomes will be analysed using mixed-effects models, including group, time, and group-by-time interaction. Categorical secondary outcomes will be analysed using chi-square test or Fisher's exact test, as appropriate. Missing primary outcome data will be handled by multiple imputation when the proportion of missing data is non-negligible and the missing-at-random assumption is considered reasonable. Complete-case analysis and per-protocol analysis will be conducted as sensitivity analyses to evaluate the robustness of the primary findings. As this is an exploratory pilot trial, secondary outcome analyses will be considered exploratory. All tests will be two-sided, with *P* < 0.05 considered statistically significant.

### 2.12 Data management

Investigators will comply with relevant regulations on source-document retention and access and will ensure that all data recorded in case report forms (CRFs) and study reports are accurate, complete, legible, timely, and traceable. Data entry will follow three principles: timeliness, with source documents and CRFs completed within 24 h whenever feasible; completeness, with all protocol-required variables collected for each participant; and accuracy, with trained study personnel recording data according to standardized procedures and performing consistency checks. After data collection, the Quality Control Committee will verify data accuracy and consistency. After project acceptance, all research data will be transferred to the Research Management Office of the sponsoring institution and retained for 5 years after study completion.

### 2.13 Patient and public involvement

Patients and members of the public were not involved in the design of the study, the choice of outcomes, the development of the recruitment strategy, or the preparation of this protocol. When feasible, interested participants will be provided with an accessible summary of the main results after study completion.

## 3 Discussion

CMVD remains a therapeutic challenge even after diagnostic recognition, and the gap between pathophysiological classification and meaningful symptom improvement is still substantial (38, 39). This exploratory sham-controlled trial therefore focuses on a clinically relevant question: whether acupuncture can provide incremental benefit beyond stable conventional therapy in patients with persistent symptoms. This question is important because symptom burden, functional limitation, and health status remain central concerns in CMVD care, and structured patient-reported outcome measures are increasingly emphasised in mechanism-based management pathways (21, 30).

A further strength of the protocol is its multidimensional outcome framework. In CMVD, symptom relief alone may not fully reflect treatment response. Combining SAQ-based health-status assessment with endothelial and inflammation-related biomarkers may improve interpretation of early-phase trial findings and help generate hypotheses regarding potential biological pathways (17, 31).

The choice of sham acupuncture as the control is methodologically important in this trial. Because key outcomes such as SAQ-based health status, angina frequency, and CGI-I are vulnerable to expectation and contextual effects, sham acupuncture may better control for non-specific effects related to practitioner attention, treatment ritual, and patient-provider interaction than usual care alone. In this study, the sham group receives the same acupoints and treatment schedule as the acupuncture group, but with non-penetrating blunt-tipped needles, in order to strengthen participant blinding and improve internal validity. However, sham acupuncture may not be completely physiologically inert, which should be considered when interpreting between-group differences.

The acupoint prescription in this trial includes SP21 (Dabao), PC6 (Neiguan), CV17 (Danzhong), BL15 (Xinshu), and BL17 (Geshu). From a traditional Chinese medicine perspective, this combination is intended to regulate qi and blood, open the chest, and unblock the collaterals. From a modern mechanistic perspective, available evidence suggests that acupuncture may influence cardiovascular function mainly through autonomic and neurohumoral modulation (40, 41). Among the selected acupoints, PC6 and CV17 are particularly relevant to chest symptoms and cardiovascular regulation; previous physiological studies have shown that stimulation at CV17 may increase the cardiac vagal component of heart rate variability, while stimulation involving PC6 may help attenuate sympathetic overactivation (42, 43). In addition, experimental work suggests that combined stimulation of PC6 and BL15 may modulate hypothalamic catecholaminergic signaling under myocardial ischemic conditions (44), and PC6 stimulation has also been associated with favorable changes in vasoactive mediators relevant to myocardial ischemia and microcirculatory regulation (45).

Several limitations should be acknowledged. First, as a single-center exploratory trial with a relatively small sample size, this study is not designed to provide definitive efficacy estimates, and the generalizability of the findings may be limited. Second, the 4-week treatment period and 4-week follow-up are suitable for capturing early symptom and biomarker changes but may not be sufficient to evaluate long-term efficacy or durability of treatment effects. In addition, NO, ET-1, and hs-CRP will be measured only at baseline and week 4, and therefore the study cannot determine whether biomarker changes persist after treatment. Third, although sham acupuncture, blinded outcome assessment, and standardized background therapy are used to reduce bias, acupuncturists cannot be fully blinded. Fourth, complete invasive coronary function testing, including CFR, IMR, and acetylcholine provocation, and contrast-based advanced imaging such as CMR-derived MPRI will not be systematically performed in all participants. This decision was made because these assessments may increase participant burden and may carry potential clinical risks, including contrast-associated kidney injury, allergic reactions, or anaphylaxis, particularly in patients with cardiovascular comorbidities. To improve feasibility and participant adherence in this preliminary pilot trial, the diagnosis of INOCA-related CMVD will be based on available clinical, angiographic, and ischemia-related evidence. If the safety and potential efficacy of acupuncture are supported by this pilot study, future larger confirmatory trials will incorporate more comprehensive coronary functional and imaging assessments, including CFR, IMR, MPRI, and acetylcholine provocation where appropriate, to validate microvascular mechanisms and improve endotype classification (39, 46, 47).

In summary, this trial is designed to provide preliminary evidence on the feasibility, safety, and potential clinical value of acupuncture for INOCA-related CMVD. If the findings are favourable, they will help inform the design of subsequent multicentre confirmatory trials and may support more standardized integrative management strategies for patients with persistent angina and suspected microvascular dysfunction.
