# Supplementary material for: Efficacy and safety of acupuncture for coronary microvascular disease: study protocol for a pilot randomized controlled trial
Source: Front Cardiovasc Med. 2026 Jul 17;13:1843651. doi: 10.3389/fcvm.2026.1843651 (PMC13423977; doi:10.3389/fcvm.2026.1843651)
Supplement: Supplementary file 1 [file Datasheet1.docx]

Supplementary Material

# Supplementary Tables

## Supplementary Table 1 SPIRIT 2025 checklist of items to address in a randomized trial protocol

| **Section / Topic** | **No** | **SPIRIT 2025 checklist item description** | **Location in manuscript** |  |
| --- | --- | --- | --- | --- |
| **Administrative information** | | |  | |
| Title and structured summary | 1a | Title stating the trial design, population, and interventions, with identification as a protocol | Title page |  |
|  | 1b | Structured summary of trial design and methods, including items from the World Health Organization Trial Registration Data Set | Abstract; Trial registration |  |
| Protocol version | 2 | Version date and identifier | Methods and analysis - Study design |  |
| Roles and responsibilities | 3a | Names, affiliations, and roles of protocol contributors | Title page; Author Contributions |  |
|  | 3b | Name and contact information for the trial sponsor | Title page |  |
|  | 3c | Role of trial sponsor and funders in design, conduct, analysis, and reporting of trial; including any authority over these activities | Funding; Conflict of Interest |  |
|  | 3d | Composition, roles, and responsibilities of the coordinating site, steering committee, endpoint adjudication committee, data management team, and other individuals or groups overseeing the trial, if applicable | Data management; Author Contributions |  |
| **Open science** | | |  | |
| Trial registration | 4 | Name of trial registry, identifying number (with URL), and date of registration. If not yet registered, name of intended registry | Trial registration; Study design |  |
| Protocol and statistical analysis plan | 5 | Where the trial protocol and statistical analysis plan can be accessed | Study design; Statistical analysis |  |
| Data sharing | 6 | Where and how the individual de-identified participant data (including data dictionary), statistical code, and any other materials will be accessible | NA |  |
| Funding and conflicts of interest | 7a | Sources of funding and other support (e.g., supply of drugs) | Funding |  |
|  | 7b | Financial and other conflicts of interest for principal investigators and steering committee members | Conflict of Interest |  |
| Dissemination policy | 8 | Plans to communicate trial results to participants, healthcare professionals, the public, and other relevant groups (e.g., reporting in trial registry, plain language summary, publication) | Patient and public involvement; Trial registration |  |
| **Introduction** | | |  | |
| Background and rationale | 9a | Scientific background and rationale, including summary of relevant studies (published and unpublished) examining benefits and harms for each intervention | Introduction; Discussion |  |
|  | 9b | Explanation for choice of comparator | Discussion |  |
| Objectives | 10 | Specific objectives related to benefits and harms | Study design |  |
| **Methods: Patient and public involvement, trial design** | | |  | |
| Patient and public involvement | 11 | Details of, or plans for, patient or public involvement in the design, conduct, and reporting of the trial | Patient and public involvement |  |
| Trial design | 12 | Description of trial design including type of trial (e.g., parallel group, crossover), allocation ratio, and framework (e.g., superiority, equivalence, non-inferiority, exploratory) | Study design |  |
| **Methods: Participants, interventions, and outcomes** | | |  | |
| Trial setting | 13 | Settings (e.g., community, hospital) and locations (e.g., countries, sites) where the trial will be conducted | Title page; Study design; Recruitment process |  |
| Eligibility criteria | 14a | Eligibility criteria for participants | Participant screening |  |
|  | 14b | If applicable, eligibility criteria for sites and for individuals who will deliver the interventions (e.g., surgeons, physiotherapists) | Interventions |  |
| Intervention and comparator | 15a | Intervention and comparator with sufficient details to allow replication including how, when, and by whom they will be administered. If relevant, where additional materials describing the intervention and comparator (e.g., intervention manual) can be accessed | Interventions; Supplementary Table 2 |  |
|  | 15b | Criteria for discontinuing or modifying allocated intervention/comparator for a trial participant (e.g., drug dose change in response to harms, participant request, or improving/worsening disease) | Written informed consent; Participant adherence; Safety evaluation |  |
|  | 15c | Strategies to improve adherence to intervention/comparator protocols, if applicable, and any procedures for monitoring adherence (e.g., drug tablet return, sessions attended) | Participant adherence |  |
|  | 15d | Concomitant care that is permitted or prohibited during the trial | Interventions |  |
| Outcomes | 16 | Primary and secondary outcomes, including the specific measurement variable (e.g., systolic blood pressure), analysis metric (e.g., change from baseline, final value, time to event), method of aggregation (e.g., median, proportion), and time point for each outcome | Outcome measures; Study schedule |  |
| Harms | 17 | How harms are defined and will be assessed (e.g., systematically, non-systematically) | Safety evaluation |  |
| Participant timeline | 18 | Time schedule of enrollment, interventions (including any run-ins and washouts), assessments, and visits for participants. A schematic diagram is highly recommended (see Figure) | Study design; Study schedule; Study flowchart |  |
| Sample size | 19 | How sample size was determined, including all assumptions supporting the sample size calculation | Sample size |  |
| Recruitment | 20 | Strategies for achieving adequate participant enrollment to reach target sample size | Recruitment process |  |
| **Methods: Assignment of interventions** | | |  | |
| Randomization |  |  |  |  |
| Sequence generation | 21a | Who will generate the random allocation sequence and the method used | Randomization, allocation concealment, and blinding |  |
|  | 21b | Type of randomization (simple or restricted) and details of any factors for stratification. To reduce predictability of a random sequence, other details of any planned restriction (e.g., blocking) should be provided in a separate document that is unavailable to those who enroll participants or assign interventions | Randomization, allocation concealment, and blinding |  |
| Allocation concealment  mechanism | 22 | Mechanism used to implement the random allocation sequence (e.g., central computer/telephone; sequentially numbered, opaque, sealed containers), describing any steps to conceal the sequence until interventions are assigned | Randomization, allocation concealment, and blinding |  |
| Implementation | 23 | Whether the personnel who will enroll and those who will assign participants to the interventions will have access to the random allocation sequence | Randomization, allocation concealment, and blinding |  |
| Blinding | 24a | Who will be blinded after assignment to interventions (e.g., participants, care providers, outcome assessors, data analysts) | Randomization, allocation concealment, and blinding |  |
|  | 24b | If blinded, how blinding will be achieved and description of the similarity of interventions | Randomization, allocation concealment, and blinding; Interventions; Additional outcomes; Supplementary Table 2 |  |
|  | 24c | If blinded, circumstances under which unblinding is permissible, and procedure for revealing a participant’s allocated intervention during the trial | Randomization, allocation concealment, and blinding |  |
| **Methods: Data collection, management, and analysis** | | |  | |
| Data collection methods | 25a | Plans for assessment and collection of trial data, including any related processes to promote data quality (e.g., duplicate measurements, training of assessors) and a description of trial instruments (e.g., questionnaires, laboratory tests) along with their reliability and validity, if known. Reference to where data collection forms can be accessed, if not in the protocol | Outcome measures; Safety evaluation; Data management |  |
|  | 25b | Plans to promote participant retention and complete follow-up, including list of any outcome data to be collected for participants who discontinue or deviate from intervention protocols | Participant adherence; Outcome measures |  |
| Data management | 26 | Plans for data entry, coding, security, and storage, including any related processes to promote data quality (e.g., double data entry; range checks for data values). Reference to where details of data management procedures can be accessed, if not in the protocol | Data management |  |
| Statistical methods | 27a | Statistical methods used to compare groups for primary and secondary outcomes, including harms | Statistical analysis; Safety evaluation |  |
|  | 27b | Definition of who will be included in each analysis (e.g., all randomized participants), and in which group | Statistical analysis |  |
|  | 27c | How missing data will be handled in the analysis | Statistical analysis |  |
|  | 27d | Methods for any additional analyses (e.g., subgroup and sensitivity analyses) | Statistical analysis |  |
| **Methods: Monitoring** | | |  | |
| Data monitoring committee | 28a | Composition of data monitoring committee (DMC); summary of its role and reporting structure; statement of whether it is independent from the sponsor and funder; conflicts of interest and reference to where further details about its charter can be found, if not in the protocol. Alternatively, an explanation of why a DMC is not needed | NA |  |
|  | 28b | Explanation of any interim analyses and stopping guidelines, including who will have access to these interim results and make the final decision to terminate the trial | NA |  |
| Trial monitoring | 29 | Frequency and procedures for monitoring trial conduct. If there is no monitoring, give explanation | Data management; Author Contributions |  |
| **Ethics** | | |  | |
| Research ethics approval | 30 | Plans for seeking research ethics committee/institutional review board approval | Study design; Ethics statement |  |
| Protocol amendments | 31 | Plans for communicating important protocol modifications to relevant parties | NA |  |
| Consent or assent | 32a | Who will obtain informed consent or assent from potential trial participants or authorized proxies, and how | Study design; Written informed consent |  |
|  | 32b | Additional consent provisions for collection and use of participant data and biological specimens in ancillary studies, if applicable | NA |  |
| Confidentiality | 33 | How personal information about potential and enrolled participants will be collected, shared, and maintained in order to protect confidentiality before, during, and after the trial | Written informed consent; Ethics statement |  |
| Ancillary and post-trial care | 34 | Provisions, if any, for ancillary and post-trial care, and for compensation to those who suffer harm from trial participation | Written informed consent; Safety evaluation |  |

*We strongly recommend reading this checklist in conjunction with the SPIRIT 2025 Explanation and Elaboration and the SPIRIT 2025 Expanded Checklist for important clarifications on all the items. We also recommend reading relevant SPIRIT extensions. See [www.consort-spirit.org](http://www.consort-spirit.org)

Citation: Chan A-W, Boutron I, Hopewell S, Moher D, Schulz KF, et al. SPIRIT 2025 statement: updated guideline for protocols of randomised trials. BMJ 2025;389:e081477. <https://dx.doi.org/10.1136/bmj-2024-081477>

© 2025 Chan A-W et al. This is an Open Access article distributed under the terms of the Creative Commons Attribution License (<https://creativecommons.org/licenses/by/4.0/>), which permits unrestricted use, distribution, and reproduction in any medium, provided the original work is properly cited.

**Supplementary Table 2 STRICTA 2010 checklist of information to include when reporting interventions in a clinical trial of acupuncture (Expansion of Item 5 from CONSORT 2010 checklist)**

| **Item** | **Detail** |
| --- | --- |
| **1. Acupuncture rationale** | 1a) Style of acupuncture  - Traditional Chinese Medicine-style manual body acupuncture**.** |
|  | 1b) Reasoning for treatment provided, based on historical context, literature sources, and/or consensus methods, with references where appropriate  - Based on Traditional Chinese Medicine theory for CMVD with qi deficiency and blood stasis, and supported by previous evidence on acupuncture for angina, endothelial dysfunction, autonomic regulation, and inflammation. |
|  | 1c) Extent to which treatment was varied  - Standardized treatment was used for all participants in the acupuncture group; no individualized modification was allowed. |
| **2. Details of needling** | 2a) Number of needle insertions per subject per session (mean and range where relevant) - Nine needles will be used in each session. |
|  | 2b) Names (or location if no standard name) of points used (uni/bilateral)   - The acupoints used are SP21 (bilateral), PC6 (bilateral), BL15 (bilateral), BL17 (bilateral), and CV17 (unilateral). |
|  | 2c) Depth of insertion, based on a specified unit of measurement, or on a particular tissue level - Insertion depth will be approximately 0.5-1 cun for SP21 and PC6, 0.5-0.8 cun for BL15 and BL17, and 0.3-0.5 cun for CV17. |
|  | 2d) Response sought (e.g. de qi or muscle twitch response) - Deqi will be sought in the acupuncture group by manual needle manipulation, typically producing sensations such as soreness, numbness, distension, or heaviness. No deqi will be sought in the sham acupuncture group. |
|  | 2e) Needle stimulation (e.g. manual, electrical) - Manual stimulation will be applied in the acupuncture group. No specific tonification or sedation method will be prespecified. No additional routine manipulation will be performed during needle retention unless needed to maintain deqi according to the standardized procedure. |
|  | 2f) Needle retention time - Needles will be retained for 20 minutes in each session. |
|  | 2g) Needle type (diameter, length, and manufacturer or material) - Acupuncture group: disposable sterile stainless-steel needles, 0.30 mm × 40 mm (Huatuo, Suzhou, China). Sham group: blunt-tipped sham needles, 0.30 mm × 25 mm (Huatuo, Suzhou, China). |
| **3. Treatment regimen** | 3a) Number of treatment sessions - A total of 12 sessions will be administered. |
|  | 3b) Frequency and duration of treatment sessions - Each session will last 20 minutes. Treatment will be administered three times per week, every other day, for 4 consecutive weeks. |
| **4. Other components of treatment** | 4a) Details of other interventions administered to the acupuncture group (e.g. moxibustion, cupping, herbs, exercises, lifestyle advice) - Both groups will receive stable guideline-directed conventional medical therapy. No additional study-specific co-interventions will be provided. |
|  | 4b) Setting and context of treatment, including instructions to practitioners, and information and explanations to patients - The trial will be conducted at Guang’anmen Hospital, China Academy of Chinese Medical Sciences, a single tertiary hospital in China. Participants will be treated in the sitting position after routine skin disinfection. To minimize visual cues, participants will be positioned so that the needling procedure is not in their direct line of sight. For forearm and anterior chest acupoints, an opaque drape or screen will be used where feasible, and participants will be instructed not to observe needle insertion or removal. Written informed consent will be obtained before enrollment and before any study-specific procedure. |
| **5. Practitioner background** | 5) Description of participating acupuncturists (qualification or professional affiliation, years in acupuncture practice, other relevant experience)  - All acupuncture procedures will be performed by licensed traditional Chinese medicine practitioners certified by the National Health Commission of the People’s Republic of China, each with at least 2 years of clinical experience in acupuncture. |
| **6. Control or comparator interventions** | 6a) Rationale for the control or comparator in the context of the research question, with sources that justify this choice - Sham acupuncture was selected to better control for non-specific effects related to practitioner attention, treatment ritual, and patient expectancy, while maintaining the same conventional medical therapy in both groups. The sham group receives the same acupoints, treatment frequency, session duration, and treatment course as the acupuncture group, but with non-penetrating blunt-tipped needles to minimize specific needling stimulation and strengthen participant blinding. |
|  | 6b) Precise description of the control or comparator. If sham acupuncture or any other type of acupuncture-like control is used, provide details as for Items 1 to 3 above. - The sham group will receive the same schedule, setting, acupoint locations, participant position, verbal instructions, session duration, and conventional medical therapy as the acupuncture group. Blunt-tipped sham needles will touch the skin surface through adhesive pads without penetrating the skin or subcutaneous tissue, and deqi will not be sought. Contact pressure will be kept light and as consistent as possible across sessions. The sham needles will have a similar external appearance to real needles, and opaque draping or screening will be used where feasible to reduce visual differences between groups. |

Note: This checklist, which should be read in conjunction with the explanations of the STRICTA items provided in the main text, is designed to replace CONSORT 2010’s item 5 when reporting an acupuncture trial.
